# Protocol for the Locomotor Experience Applied Post-stroke (LEAPS) trial: a randomized controlled trial

**DOI:** 10.1186/1471-2377-7-39

**Published:** 2007-11-08

**Authors:** Pamela W Duncan, Katherine J Sullivan, Andrea L Behrman, Stanley P Azen, Samuel S Wu, Stephen E Nadeau, Bruce H Dobkin, Dorian K Rose, Julie K Tilson, The LEAPS Investigative Team

**Affiliations:** 1 Division of Doctor of Physical Therapy, Department of Community and Family Medicine, Duke University, Durham, North Carolina, USA; 2 Center for Clinical Health Policy Research, Duke University, Durham, North Carolina, USA; 3 Center for Aging, Duke University, Durham, North Carolina, USA; 4 Department of Biokinesiology and Physical Therapy, University of Southern California, Los Angeles, California, USA; 5 Department of Physical Therapy, Brooks Center for Rehabilitation Studies, University of Florida, Gainesville, Florida, USA; 6 Department of Veteran Affairs Brain Rehabilitation Research Center, Gainesville, Florida, USA; 7 Biostatistics Division, Department of Preventive Medicine, University of Southern California, Los Angeles, California, USA; 8 Department of Epidemiology and Health Policy Research, University of Florida, Florida, USA; 9 Geriatric Research, Education and Clinical Center, Malcom Randall VA Medical Center, Gainesville, Florida, USA; 10 Department of Neurology, University of California, Los Angeles, California, USA; 11 Department of Aging and Geriatric Research, University of Florida, Gainesville, Florida, USA

## Abstract

**Background:**

Locomotor training using body weight support and a treadmill as a therapeutic modality for rehabilitation of walking post-stroke is being rapidly adopted into clinical practice. There is an urgent need for a well-designed trial to determine the effectiveness of this intervention.

The objective of the Locomotor Experience Applied Post-Stroke (LEAPS) trial is to determine if there is a difference in the proportion of participants who recover walking ability at one year post-stroke when randomized to a specialized locomotor training program (LTP), conducted at 2- or 6-months post-stroke, or those randomized to a home based non-specific, low intensity exercise intervention (HEP) provided 2 months post-stroke. We will determine if the timing of LTP delivery affects gait speed at 1 year and whether initial impairment severity interacts with the timing of LTP. The effect of number of treatment sessions will be determined by changes in gait speed taken pre-treatment and post-12, -24, and -36 sessions.

**Methods/Design:**

We will recruit 400 adults with moderate or severe walking limitations within 30 days of stroke onset. At two months post stroke, participants are stratified by locomotor impairment severity as determined by overground walking speed and randomly assigned to one of three groups: (a) LTP-Early; (b) LTP-Late or (c) Home Exercise Program -Early. The LTP program includes body weight support on a treadmill and overground training. The LTP and HEP interventions are delivered for 36 sessions over 12 weeks.

Primary outcome measure include successful walking recovery defined as the achievement of a 0.4 m/s gait speed or greater by persons with initial severe gait impairment or the achievement of a 0.8 m/s gait speed or greater by persons with initial moderate gait impairment.

LEAPS is powered to detect a 20% difference in the proportion of participants achieving successful locomotor recovery between the LTP groups and the HEP group, and a 0.1 m/s mean difference in gait speed change between the two LTP groups.

**Discussion:**

The goal of this single-blinded, phase III randomized clinical trial is to provide evidence to guide post-stroke walking recovery programs.

**Trial registration:**

NCT00243919.

## Background

Of the 730,000 individuals who will survive a stroke each year, 73% will have residual disability [1]. Locomotor ability is an important factor in determining the degree of physical disability after stroke [2]. The impact of stroke on walking is significant, with only 37% of stroke survivors able to walk after the first week post-stroke [3-5]. Sixty to eighty percent of individuals who achieve independent ambulation, walk at speeds less than 0.8 m/s, which is insufficient to function effectively in the community [6]. Significant residual deficits in balance persist with a 73% incidence of falls among individuals with mild to moderate impairment 6 months post-stroke [7,8]. Walking and balance deficits contribute substantially to long-term disability post-stroke. In the Northern Manhattan Stroke Study, over 40% of individuals with stroke living at home required assistance with walking at 6 months. Among the 60% of individuals considered independent walkers by ADL indices, significant disability due to limitations in community ambulation skills persisted [9]. Ambulatory stroke patients experience a 4-fold increase in falls risk, and among those who fall, a 10-fold increase in hip fracture [10].

A body weight support system and treadmill (BWST) is one therapeutic modality for locomotor training that is rapidly being adopted into physical rehabilitation to improve walking after stroke. There are 20 clinical studies that have examined the efficacy of this treatment approach [11,12]. The most recently published trials of walking programs that included treadmill training in acute [13] and chronic [13-15] stroke patients reported improved gait speed compared to individuals who participated in non-specific low intensity exercise programs. However, the conclusions of the Cochrane systematic review and meta-analyses report that there is "not enough evidence from trials to determine the effect of treadmill training with or without body weight support for walking after stroke [16]."

Of 20 trials of treadmill training, only 14 are RCTs and 8 of the 14 trials had 30 or fewer participants and the maximum number of participants was 100. These trials vary substantially in training intensity (i.e., walking time, treadmill speed, and percent of weight support), frequency of training sessions per week, total number of sessions, timing of training (acute, subacute or chronic), and locomotor impairment severity (non-ambulators to community ambulators). Due to the differences between existing studies, there is lack of evidence concerning (1) when this intervention is most effective post-stroke (e.g. acute, subacute, chronic), (2) the effect of locomotor impairment severity on achieving clinically significant outcomes and, (3) the optimal duration for the locomotor training intervention. The studies have also failed to consistently evaluate parameters of training, such as speed. Yet, recent studies have consistently shown that treadmill training (with or without BWS) at higher speeds (i.e., higher intensity) is more effective at improving walking after stroke than training at slower speeds [17-19]. Given the current heterogeneity in study protocols and the inconclusive results of systematic analysis of the trials, the Cochrane review states that there is an "urgent need for well-designed large-scale studies to evaluate the effects of treadmill training and body weight support on walking after stroke [11]."

The Locomotor Experience Applied Post-Stroke (LEAPS) trial is a 5-year, phase-III, single-blind, 5-site, randomized controlled trial (RCT) to determine if there is a difference between treatment groups in the proportion of participants who at one year post-stoke successfully recover walking ability, as defined by gait speed. The intervention groups under study are: (1) a specialized locomotor training program (LTP) that includes use of body weight support and a treadmill as a rehabilitation modality provided 2 months post-stroke (LTP-early), or (2) 6 months post-stroke (LTP-late), and (3) a non-specific, low intensity home-based exercise (HEP) intervention provided 2 months post-stroke.

The study is designed as a definitive RCT with the primary outcome being successful recovery of walking. Successful recovery of walking is defined as having achieved a 0.4 m/s gait speed or greater for persons with initial severe gait impairment (< 0.4 m/s), or as having achieved a 0.8 m/s gait speed or greater for persons with initial moderate gait impairment (≥ 0.4 m/s – < 0.8 m/s). A clinically significant difference will be defined as a greater than 20% difference in the proportion of participants who achieve successful recovery.

We hypothesize that both the LTP-early and LTP-late groups will have more success than the home exercise group. We will also determine if the timing of LTP delivery (early vs. late) affects the improvement in gait speed at 1 year and whether initial locomotor impairment severity interacts with the timing of LTP delivery. We anticipate that earlier intervention will be more efficacious because it is conducted at a time when the endogenous neuroplastic processes that follow stroke are more active [20]. However, we also suspect that there will be a timing by severity interaction effect: participants with modest deficits may be able to take full advantage of the neuroplastic effect and thereby benefit more from early LTP, whereas participants with more severe deficits may have to experience more extended spontaneous recovery to be able to take full advantage of the LTP, thereby benefiting more from late LTP. The LTP and HEP interventions are delivered for 36 sessions over 12 weeks to 16 weeks. The effect of number of treatment sessions will be determined by changes in gait speed measured pre-treatment, post-12, post-24, and post-36 sessions. We anticipate that more treatments will yield greater benefit, but that there may be a severity interaction effect here also: participants with milder deficits may be more likely to asymptotically approach maximum therapeutic benefit in less than 36 sessions, whereas participants with more severe deficits, by virtue of a slower rate of improvement, may continue to improve throughout the entire 36-session course.

## Methods/Design

All procedures conducted during this trial with human participants were carried out in compliance with federal and institutional ethical standards and in compliance with the Helsinki Declaration. All research procedures were approved by an Institutional Review Board at each participating site: Duke University Health System Institutional Review Board (protocol #9500-07-3RO), University of Florida Health Science Center Institutional Review Board (protocol #262-2005), University of Southern California Health Sciences Campus Institutional Review Board (protocol # HS-05-00365), Brooks Center for Rehabilitation Studies approved by the University of Florida Health Science Center Institutional Review Board (protocol #262-2005), Centinela Freeman Regional Medical Center approved by Western Institutional Review Board (study # 1077658), Florida Hospital Institutional Review Board (protocol # 2005.09.11), Long Beach Memorial Hospital Memorial Health Service Research Council (protocol #286-05) and Sharp Rehabilitation Center Sharp HealthCare Institutional Review Board (protocol #050896).

### Type of Design

**Figure 1:**
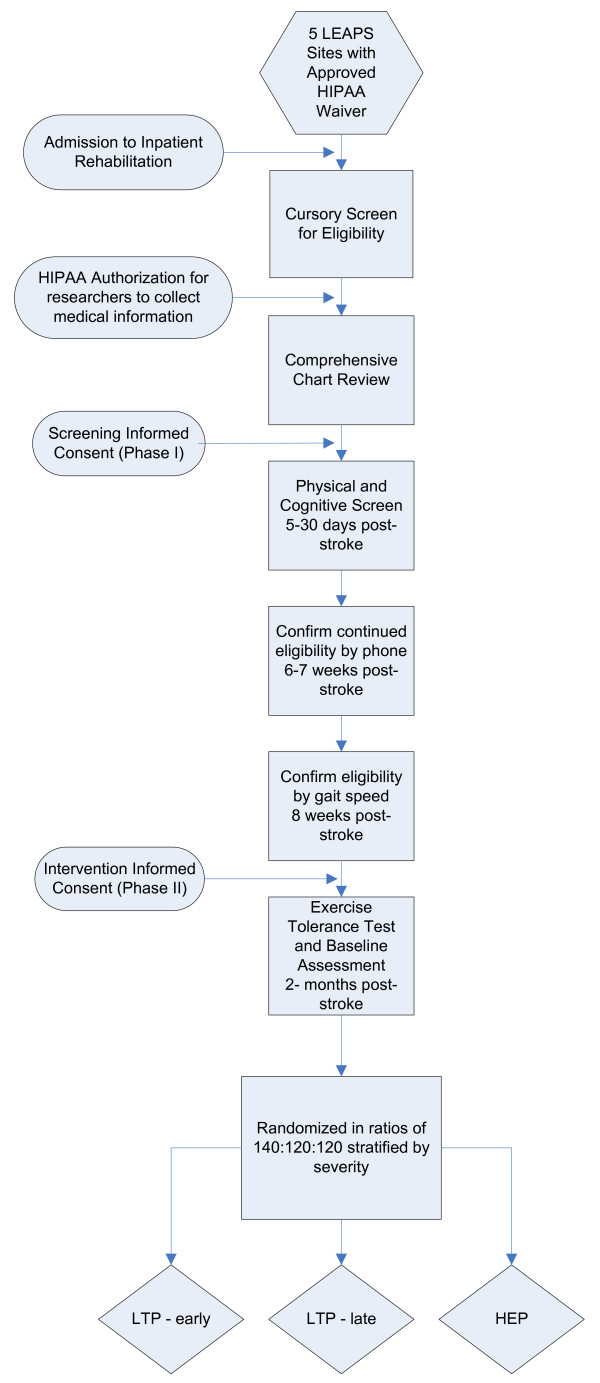
Sequence of participant screening, enrollment and randomization.

This is a three arm, single blinded, phase III randomized controlled trial of a locomotor training program provided at 2 months post-stroke or 6 months post-stroke versus a home exercise program provided at 2 months post-stroke. Participants are randomized to the three intervention groups at 2 months post-stroke. The primary outcome is the proportion of participants who successfully recover walking one year post-stroke. The sequence from screening, enrollment and to randomization is represented in Figure 1.

### Study Enrollment

Individuals post- stroke over the age of 18 are recruited 5–30 days post-stroke from five clinical sites. The five clinical intervention sites: Brooks Rehabilitation Hospital in Jacksonville, Florida; Centinela Freeman Regional Medical Center in Inglewood, California; Florida Hospital in Orlando, Florida; Long Beach Memorial Hospital in Long Beach, California and Sharp Rehabilitation Center in San Diego, California. Each site is expected to recruit approximately 80 participants.

### Accessing and Collecting Personal Health Information

A two part process for accessing and collecting personal health information ensures protection of potential participants' rights under the Health Information Portability and Accountability Act (HIPAA). First, each site obtains a HIPAA Waiver from the site's Institutional Review Board or the Health Information Safety Committee. The HIPAA Wavier allows a cursory screening of patient's chart to identify criteria that are exclusionary: diagnosis, age, medical comorbidities, life expectancy and distance of discharge residence from clinical site. No personal health information is recorded at this stage. Second, if this cursory chart screen is passed, potential participants are approached by a site research team member to explain the study and to request a HIPAA authorization. This HIPAA authorization permits study staff to fully review the patient's chart to determine study eligibility and to collect and record personal health information. In the event that a potential participant declines at this point, no identifiable information is kept on that participant. However, we do report the reasons for exclusion as either (1) did not meet primary diagnosis of stroke; (2) did not meet age criteria (3) had other major medical comorbidity, (4) life expectancy less than one year, (5) plan to be discharged greater than 50 miles from intervention site, or 6) declines participation.

### Informed Consent

Informed consent for study participation and enrollment occurs in two phases: (1) a screening informed consent to participate in a physical screen to determine study eligibility is obtained after the HIPAA authorized chart review and (2) an intervention informed consent is obtained at 8 weeks post-stroke that provides consent for an exercise tolerance test, baseline and follow-up assessments, and intervention. Participants are informed that successful completion of the exercise tolerance test is required prior to randomization to an intervention group.

### Screening Process

Table 1 summarizes the consent, screening processes, and assessments that are conducted at 5–30 days, 6–8 weeks, and at 2 months post-stroke, prior to randomization.

**Table 1:** Timeline for Consent, Screening Processes and Assessments up to Randomization

**Time Point**	**Activity**	**Specific Tests and Procedures**
**5–30 days post-stroke**	HIPAA Waiver Screen	Chart review for diagnosis, age, medical co-morbidities, life expectancy, discharge distance from clinical site
	HIPAA Authorization	Patient grants permission for study staff to complete a comprehensive chart review
	Chart Review	Demographics collected, inclusion and exclusion criteria reviewed
	Screening Informed Consent	Study staff review screening procedures, risks and benefits
	Participant/family interview	Study staff determine:
		• Pre-morbid functional status
		• Enrollment in another clinical trial
		• Ability to attend therapy 3×/week
	Physical and Cognitive	Study staff complete tests:
	Screen	• 3-Step Command
		• Sitting Balance
		• Ability to walk 10 feet
		• 10 meter walk test (when able)
		• Functional Ambulation Category
		• Fugl-Meyer Motor and Sensory Tests
		• PHQ-9 Depression Scale
		• ROM of Lower Extremities
		• Contracture Assessment of Lower Extremities
		• MMSE
		• SF-36 Physical Function
		• Pre-morbid Barthel Index
		• Orpington Prognostic Scale
		• Modified Rankin Index
	MD Approval	Confirmation by treating physician that patient meets inclusion/exclusion criteria and is safe for the study
**6 – 7 weeks post-stroke**	6-Week Phone Contact	Interview confirms continued eligibility
**8 weeks post-stroke**	Gait Velocity Re-check	10 meter walk test – confirm velocity <.8 m/s
	Intervention Informed Consent	Study staff review exercise tolerance test and intervention procedures, risks, and benefits
	Exercise Tolerance Test	Cardiologist approves eligibility
	Lesion Analysis	Study neurologist reads MRI or CT to characterize lesion
**2 months post-stroke**	Baseline Assessment	See Table 2 for assessment tests

The purpose of the 5–30 day screening phase is to determine if the individual post-stroke is willing to participate in the LEAPS trial and meets preliminary inclusion and exclusion criteria for study participation. If the participant voluntarily provides informed consent and successfully completes all 5–30 day physical and cognitive screens, the participant's treating physician is asked to review the LEAPS inclusion and exclusion criteria and provide a letter of support for inclusion in the study. At 6 weeks post-stroke, a phone contact is made by a study team member to confirm interest and to determine continued eligibility. The participant or a proxy family member is asked about the participant's current health status, recent hospitalizations, changes in residential status (i.e., moved to a nursing home) and whether or not they wish to continue in the trial. Specific questions are asked regarding possible cardiac conditions, recurrent stroke, possible fractures and new medical events that may limit activities of daily living. At 8 weeks post-stroke, the participant returns to the study site to confirm that gait speed is < 0.8 m/s, provide the phase 2 informed consent, and complete the exercise tolerance test. The exercise tolerance test, the baseline assessments, and the analysis of the most recent available CT or MRI scan are completed prior to randomization. The CT or MRI scan is reviewed by one of the study's consulting neurologists.

When the participant is a non-English speaker, an interpreter, either a family member, friend, or professional who can communicate with the participant, is available during screening, assessment and intervention sessions

### Inclusion and Exclusion Criteria

#### Stroke Diagnosis

Participants are individuals with recent onset of ischemic or hemorrhagic stroke. For purposes of inclusion in this study, a stroke is defined according to the World Health Organization definition as, "a rapid onset event of vascular origin reflecting a focal disturbance of cerebral function, excluding isolated impairments of higher function and persisting longer than 24 hours [21]." Stroke diagnosis is confirmed by CT or MRI scan or, if scan is not available, by clinical criteria.

#### Inclusion Criteria

Inclusion criteria for the trial include (1) age ≥ 18, (2) stroke within 30 days, (3) residual paresis in the lower extremity (Fugl-Meyer lower extremity score < 34), (4) ability to sit unsupported for 30 seconds, (5) ability to walk at least 10 feet with maximum 1 person assist, (6) ability to follow a three-step command, (7) physician approval for patient participation, (8) provision of informed consent, (9) self-selected 10 meter gait speed less than 0.8 m/s at the 2 month assessment, (10) successful completion of the bicycle ergometer exercise tolerance test at the 2 month assessment (see below) and, (11) living in the community at 2 months post-stroke or if they are living in a nursing home they are expected to be discharged to home and will be able to travel to the intervention site to participate in the LTP program or will be able to receive the HEP program in the nursing home facility.

#### Exclusion Criteria

Exclusion criteria for participation in this study include: (1) Lived in nursing home prior to stroke, (2) Unable to ambulate at least 150 feet prior to stroke, or intermittent claudication while walking less than 200 meters, and (3) Serious cardiac conditions (hospitalization for myocardial infarction or heart surgery within 3 months, history of congestive heart failure, documented serious and unstable cardiac arrhythmias, hypertrophic cardiomyopathy, severe aortic stenosis, angina or dyspnea at rest or during activities of daily living). Anyone meeting New York Heart Association criteria for Class 3 or Class 4 heart disease is excluded. Those who have undergone coronary artery bypass grafts (CABG) or have had mitral valve replacements within the last 3 months are excluded if their participation is not approved by 2 physicians. One of the physicians making the determination must be a cardiothoracic surgeon and the other must be either a cardiologist or the participant's primary care physician.

Additional exclusion criteria include: (4) History of serious chronic obstructive pulmonary disease or oxygen dependence, (5) Severe weight bearing pain, (6) Preexisting neurological disorders such as Parkinson's disease, Amyotrophic Lateral Sclerosis (ALS), Multiple Sclerosis (MS), dementia, or previous stroke with residual motor deficits, (7) History of major head trauma, (8) Lower extremity amputation, (9) Non-healing ulcers of a lower extremity, (10) Renal dialysis or end stage liver disease, (11) Legal blindness or severe visual impairment, (12) A history of significant psychiatric illness defined by diagnosis of bipolar affective disorder, psychosis, schizophrenia or medication refractory depression, (13) Life expectancy less than one year, (14) Severe arthritis or orthopedic problems that limit passive ranges of motion of lower extremity (knee flexion contracture of > 10°, knee flexion ROM < 90°, hip flexion contracture > 25°, and ankle plantar flexion contracture > 15°, (15) History of sustained alcoholism or drug abuse in the last six months, and (16) major post-stroke depression as indicated by a Patient Health Questionnaire (PHQ)-9 score of greater than 10 in the absence of documented management of the depression by a health care provider (either anti-depressant medication or psychotherapy), (17) History of pulmonary embolism within 6 months, (18) Uncontrollable diabetes with recent weight loss, diabetic coma, or frequent insulin reactions, (19) Severe hypertension with systolic blood pressure greater than 200 mmHg and diastolic blood pressure greater than 110 mmHg at rest, that can not be medically controlled into the resting range of 180/100 mmHg, (20) Previous or current enrollment in a clinical trial to enhance stroke motor recovery, (21) Residence more than 50 miles from the training sites, (22) Inability to travel 3 times per week for outpatient training programs; and (23) Intracranial hemorrhage related to aneurysmal rupture or an arteriovenous malformation (hemorrhagic infarctions will not be excluded).

### Exercise Tolerance Test

The bicycle ergometry protocol used successfully in the Kansas City Post-Acute Stroke Study[22] is used to assess exercise tolerance in this trial. This exercise tolerance test is conducted in cardiac stress test laboratories at the respective sites. When sitting on the bicycle, the participant's resting diastolic BP must be less than 100, systolic BP less than 180, and heart rate less than 100 to begin the testing session. The bicycle ergometry protocol requires pedaling at 40–60 RPM with a workload increase of 10 watts (from initial 0 watts) per minute. Testing continues until maximal effort is achieved. Maximal effort is defined as achievement of 90% maximal predicted heart rate (220-age). The test is terminated prior to achieving 90% maximum heart rate if the person experiences onset of limiting symptoms or meets criteria for halting the test due to blood pressure, oxygen desaturation or abnormal electrocardiographic changes. Participants will be asked to report their rate of perceived exertion, using the Borg Scale, at 10 W increments. During the graded exercise test, blood pressure readings are obtained every minute. Heart rate is obtained from the 12-lead EKG. In patients who are taking beta blockers the test is terminated if the participant reports a Borg score of greater than 18.

### Assessments

#### Methods and Timing of Assessments

Measures selected have established reliability and validity and are captured according to standardized protocols that are defined in a manual of operations Per diem therapists who are unaware of treatment assignment conduct 2 month baseline assessments and all follow-up assessments.

Two month baseline assessments are conducted prior to randomization. Participants are subsequently educated to refrain from discussing assignment group during evaluations. In addition, posters are placed in the evaluation labs to remind patients not to reveal their group assignment. To determine the effectiveness of our single blinded assessments, we ask both the per diem therapist-evaluators and the participants to complete a brief assessment to determine if group assignment was revealed during evaluations. All follow-up measures are performed at times and places where training does not occur.

All participants undergo evaluation of primary and secondary outcome measures at baseline (2 months post-stroke), 6 months and 12 months post-stroke. In addition, during the intervention phase assessments of gait speed, walking endurance, and amount of daily walking after 12, 24, and 36 training sessions are recorded. Table 2 provides a list of the measures and the timing of assessments.

**Table 2:** Summary of baseline and follow-up assessments for all subjects at each time point

**Assessment**	**Baseline 2 months**	**6 months**	**12 months**	Post-12 sessions*	Post-24 sessions*	Post-36 sessions*
10-meter Walk (orthotic and assistive device)	X	X	X	X	X	X
Functional Ambulation Category Level	X	X	X	X	X	X

6-minute Walk Test	X	X	X	X	X	X
Step Activity Monitor	X	X	X	X	X	X
Fugl-Meyer Motor and Sensory	X	X	X			
Berg Balance Scale	X	X	X			
Anthropometric Measurements	X	X	X			
Trail Making Test A and B	X	X	X			
Digit Symbol – WAIS	X	X	X			
MMSE	X	X	X			
ABC Confidence Scale	X	X	X			
Stroke Impact Scale	X	X	X			
SF-36 Physical Function	X	X	X			
Co-morbidity and Functional Impact Index	X	X	X			
PHQ-9 Depression Scale	X	X	X			
Medication Information	X	X	X			

Past Hospitalization	X	X	X			
Modified Rankin Scale	X	X	X			

• These assessments are completed between the baseline and 6 month assessment for LTP-Early and HEP participants and between the 6 month and 12 months assessments for the LTP-Late group.

#### Measures

##### Primary Measure of Walking: Gait Speed

Gait speed at one year has been selected as the primary outcome measure for this study and is measured during a 10-meter walk. Individuals are given a 3 meter warm-up distance for walking, preceding the 10 meter distance and 3 meters beyond the 10 meters to continue walking. The time that it takes to traverse the 10 meters at the participant's usual pace is recorded. Increasing walking speed is critical for community ambulation and measures reserve and adaptability [2] Gait speed is related to the severity of impairment in the home and the community. Perry et al.[2] placed study participants into one of 6 functional walking categories to relate social outcomes of patients with gait impairments. The most significant difference between groups was preferred walking speed, with mean speeds ranging from < 0.4 m/s for household walkers, 0.4 – 0.8 m/s for limited community walkers, and > 0.8 m/s for community walkers. Schmid, Duncan, Studenski et al. recently demonstrated that transitioning from one ambulation class to another was correlated with improvement in physical functioning and quality of life [23]. They concluded that outcomes assessments based on transitions within a mobility classification scheme that is rooted in gait velocity yields meaningful indicators of clinical benefit. Additionally, in a geriatric population Studenski, Duncan et al. revealed that gait speed < 0.6 m/s is a strong indicator of future health care utilization and functional decline [24]. Excellent (ICCs ≥ .97) interrater and intrarater reliability estimates for self-paced timed forward walking using a stopwatch have been reported [17].

#### Secondary Measures of Walking

In addition to walking speed, the distance a person can walk and the amount of daily walking that a person is able and willing to do are strong indicators of his or her health and condition [17]. Thus, LEAPS also obtains a 6-minute walk test and monitors community ambulation using a step activity monitor. The six-minute walk is a measure that was originally developed to assess cardiopulmonary function [25,26], but it has been used extensively as an endurance measure in the elderly and in individuals with stroke [17]. Participants perform the 6-minute walk using a previously standardized protocol [17]. In this test, participants are allowed 6 minutes to walk as far as they can at their usual pace. During the 6-minute walk test, the participants use their customary assistive devices and orthotics. The distance they cover in 6 minutes is recorded.

To measure the amount of self-selected walking over an extended period of time, LEAPS uses a Step Activity Monitor (SAM). The device is safe, highly accurate, unobtrusive for the wearer, capable of continuously recording data in short time increments, and capable of withstanding daily home and community use [27]. The device has a 99% accuracy for recording steps with a variety of gait patterns [27-29]. The device has also been successfully tested in monitoring ambulatory activity in persons with mild to moderate impairments post-stroke [30]. High test re-test reliability (r = 0.81) has been demonstrated across three separate 4-day weekday recording epochs conducted within a 3-week period in persons with chronic stroke [30]. A demonstration and written instructions are provided to participants in appropriate use of the device. Follow-up phone calls by the therapists are made during the 4-day intervals to enhance compliance and respond to any problems.

##### Functional Ambulation Classification

This functional ambulation classification system categorizes participants according to basic skills necessary for functional ambulation, without assessing the factor of endurance. The screening therapist uses standardized definitions to classify the participant according to one of six categories during the 6 minute walk. The participants are rated at their most independent level of function with regard to supervision or physical assistance needed from another person [31].

##### Sensory and Motor Control

###### Fugl-Meyer Motor Assessment

The Fugl-Meyer sensory motor assessment is probably the most widely known scale of motor and sensory recovery after stroke [32,33]. It is used for both clinical and research purposes. The Fugl-Meyer includes items of upper and lower extremity function that require progressively more complex movements, hand grasps, and measures of speed, coordination, light touch, and proprioception. Each item is graded on a three-point scale (0 cannot perform, 1 performs partially, and 2 performs fully). Standardized protocols for administration are followed [32].

###### Balance

Balance is measured by the Berg Balance Scale [34]. The scale consists of 14 items that require participants to maintain positions of varying difficulty and perform specific tasks such as rising from a chair and timed stepping. Each item is graded 0 to 4. A standardized protocol for administration is followed [34]. The Berg Balance Scale has been tested in a stroke population and has well-established reliability and validity [34].

##### Cognitive Measures

The Mini Mental Status Examination (MMSE) is used to assess cognitive function [35-37]. The Digit Symbol – Coding from the WAIS III [38] and Trail Making Test A and B [39,40] are additional cognitive measures.

For Digit Symbol-Coding the participant copies symbols that are paired with numbers. Using a key, the participant draws each symbol under its corresponding number. The participant's score is determined by the number of symbols correctly drawn within the 120-second time limit.

The Trails A measure requires the patient to sequence numbers in a specified manner under the pressure of a time. Trails B requires the patient to alternate between two sets of different information while under the pressures of time.

##### Balance Efficacy

The Activities-Specific Balance (ABC) Scale [41] is a self-report measure and is used to assess perceived efficacy (self-reported confidence) in maintaining balance while performing a number of activities common in community-dwelling older adults such as bending, reaching, and walking both inside and outside the home. This measure has good reliability and internal consistency [41].

##### Depression Screen

The Patient Health Questionnaire nine-item depression scale (PHQ-9) is used to measure depression. The PHQ-9 is increasingly used in primary care and other medical populations [42-44]. Its usefulness as a depression screening and diagnostic instrument has been recently established for individuals with stroke [45]. The PHQ-9 is a summed scale with scores ranging from 0 (no depressive symptoms) to 27 (all symptoms occurring daily). Cutoff points of 5, 10, 15, 20 represent the thresholds for mild, moderate, moderately severe, and severe depression. A PHQ-9 score of ≥ 10 in individuals with stroke has 91% sensitivity and 89% specificity for major depression [46].

##### Stroke Specific Disability Measure, the Stroke Impact Scale

The Stroke Impact Scale (SIS) is a comprehensive and psychometrically robust stroke-specific outcome measure [46-48]. The SIS was developed from the perspective of patients, caregivers, and health professionals with stroke expertise [48]. In version 3.0, there are eight domains and 59 items. The domains are: strength, hand function, activities of daily living/instrumental activities of daily living, mobility, communication, emotion, memory, and thinking, and social participation [49]. A comparison of the SIS (physical and social functioning domains) and the Short Form-36 (SF-36) has been completed [50]. The results of the study indicated that the SIS is better at capturing physical functioning and social well-being in patients with stroke then the SF-36 [51].

##### Other Measures

###### SF-36 Physical Function

The physical functioning portion of the SF-36 consists of 10 items and assesses the impact of a participant's health on their physical functioning. Participants are asked to answer the questions regarding activities that they might do in a typical day and whether or not their health limits these activities. This information is collected at baseline assessment and at the 6-month and 12-month assessments. During screening phase the physical functioning portion of the SF-36 is administered in relation to the participant's pre-morbid functioning at one month prior to stroke onset. The measure is used during post-randomization assessments to measure the participant's current functioning.

###### Co-Morbidity and Functional Impact Scale

A disease and symptom co-morbidity index developed and tested in a stroke population by Rigler, Studenski, Wallace, Reker and Duncan to predict post-stroke function is administered at baseline [52].

###### Anthropometric Measures

Measures of height, weight and waist circumference are taken. Height is a major indicator of general body size and bone length. It is important in the interpretation of weight. Weight is a composite measure of total body size. Strictly, this measurement is of mass rather than weight, but the latter term is well-established in the common lexicon. Waist circumference is an index of deep adipose tissue and is related to fat-free mass. These measures are taken at baseline (2 months post-stroke) 6 month and 12 month post-stroke assessments.

###### Falls

All participants are asked to keep a falls diary. Each participant is given a definition of falls and a supply of 30-day calendars on which to keep a record of falls. Each participant is given a packet of self-addressed postcards to mail if he or she experiences a fall. If a postcard is received, the participant receives a structured phone call to obtain information about the location and conditions of the fall as well as about any injuries. These methods have been utilized in previous studies by Studenski and Duncan [53,54].

###### Usual Care Intervention Logs

The purpose of the Usual Care Intervention Log is to track the amount of physical and occupational therapy LEAPS participants receive during enrollment in the trial (2 months to 12-months post-stroke) from outside sources. Participants are instructed to write in the time (in minutes) of occupational or physical therapy they receive outside of their participation in the LEAPS clinical trial on monthly calendars provided for them. They turn in a monthly calendar to the intervention therapist during the intervention period indicating any additional physical or occupational therapy during that time. When the participants are not actively enrolled in the intervention they are provided stamped, addressed envelopes to return their monthly calendar via mail to the clinical site for the duration of LEAPS follow-up. Participants are prompted to return these calendars at the monthly phone call. Participants turn in a calendar monthly, even if no occupational or physical therapy was received.

###### Medication Information

Information on the medications taken by a participant are recorded at baseline and at the 6- and 12- month assessments. Participant's are asked to bring a list, or the actual medication containers, of all over-the-counter and prescription medications that they have taken in the past week. The list of medications, the overall number of medications and whether dizziness is a side effect of any of the medications is recorded.

### Standardization of Assessments

Standardization of data collection methods is achieved through a systematic training and competency assessment program for all per diem physical therapists masked to the intervention. The assessors are introduced to each of the outcome measures in a lecture and demonstration style educational setting. This is followed by practice on volunteers (usually persons with stroke) under the supervision of the study's clinical research coordinators and site team leaders. The assessor must pass a competency-based evaluation, which is videotaped. At least 90% achievement of standardized skills must be documented for all primary outcome measures, including the Fugl-Meyer Motor and Sensory Assessment, and the Berg Balance Test. Performance of all outcome measures is reviewed and approved by the study clinical research coordinators to ensure standardization. Continual training and feedback is provided to ensure sustained quality of outcome measures.

### Randomization Methods

A total of 400 participants are randomly assigned to one of the three groups: (1) LTP-early; (2) LTP-late; or (3) HEP. Treatment allocation occurs when a participant is eligible for the intervention, baseline assessments at 2 months post-stroke are complete, and the consent for randomization has been obtained. The study coordinator registers the patient, enters the baseline data into the web-based database system, and then obtains group assignment from the data management and analysis center.

Because of concern that a simple randomization might yield significant imbalance in baseline impairment severity within the three treatment groups at each site, an adaptive treatment allocation procedure has been adopted. Participants are stratified by the baseline impairment severity rating into two strata (< 0.4 m/s and ≥ 0.4 – < 0.8 m/s), and for each severity stratum within each clinical site, they are randomly assigned to the LTP-early, LTP-late and HEP groups in the proportions of 140:140:120. As each participant is presented to the web-based system for random allocation, the system determines the difference between the true accumulations and the target distribution across the three groups. If there is imbalance, a participant is assigned to the underrepresented group with 80% probability and to the other two groups with 10% probability each. As an example, we may define imbalance as *d*_*i *_= max_*j *_|*f*_*ij *_- *F*_*ij *_| being larger than three, where *f*_*ij *_and *F*_*ij *_are the realized and expected frequencies of participants from the *i*^*th *^stratum in the *j*^*th *^treatment group within the site. Rules and cutoffs are determined by the DMAC.

### Interventions

Participants are randomized to 1 of three intervention groups, each receiving 3 treatment sessions per week for 12 to 16 weeks (36 total sessions):

1. Early Locomotor training (LTP-early) – High intensity locomotor training program that includes both walking training on a treadmill with partial body weight support and overground provided 2 months after stroke.

2. Late Locomotor training (LTP-late) – High intensity locomotor training program that includes both walking training on a treadmill with partial body weight support and overground provided 6 months after stroke.

3. Home-based exercise (HEP-early) -, Low-intensity exercise program focused on strength, balance and coordination provided in the home 2 months after stroke.

In addition to the LTP and HEP interventions, all participants receive any prescribed usual and customary care during the intervening periods. There are several reasons to allow individuals to receive usual and customary care. First, facilities may be less likely to refer clients to this trial if they believe they will lose revenues from outpatient or home health treatments. Second, participants may be inhibited from enrolling if they believe trial participation will reduce their opportunities to participate in other therapy. However, participants are required to abstain from any therapeutic intervention during the full period of their enrollment and participation that uses a treadmill or body weight support device (either over a treadmill or overground) unless under the supervision of the trial. Usual care interventions are monitored with participant self report logs.

Prior to initiating the intervention, each participant is provided a Participant's Guide for orientation to the study team, intervention, and expectations. The trainer reviews the guide content with the participant across several sessions. The guide content covers contact information for the primary PT, schedule, falls calendar, an overview of the responsibilities and role of the training team and participant, and overview of the LTP or HEP and how to make the most of gains after the program is completed. Participants are asked to identify their primary walking goal, i.e. "I want to walk my dog in the park", "I want to be able to drive my lawnmower equipment". At each training session, specific training goals are reviewed with the participant and in the context of achieving the participant's goal. As the participant's skills progress during the training, the trainers also ask the participant, "what is limiting you from achieving your goal relative to walking?". Trainers may use the response to tailor the goals and parameters of the training session.

#### Locomotor Training Program

**Figure 2:**
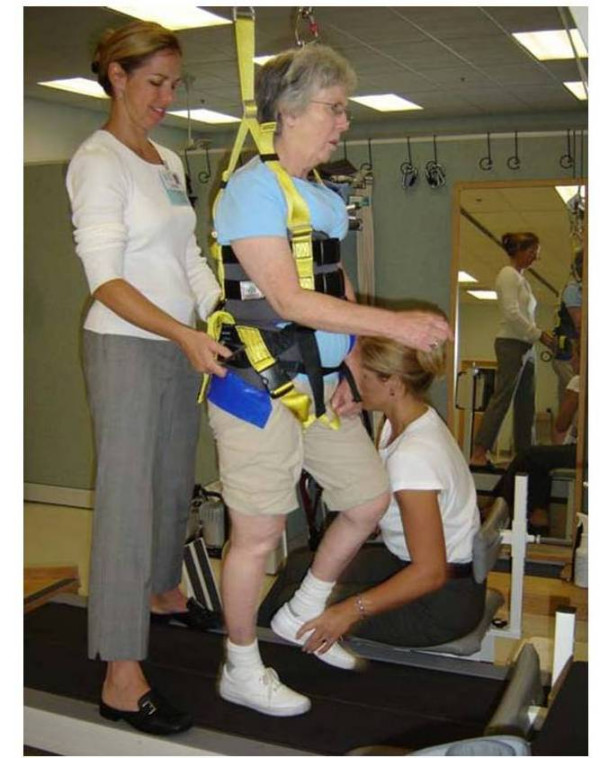
Locomotor Training Program (LTP) – Body weight support with treadmill training. Figure 2 reprinted with permission from Sage Publications [55].

**Figure 3:**
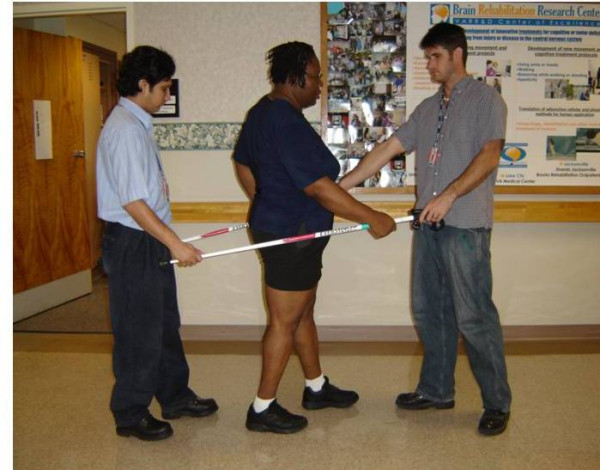
Locomotor Training Program (LTP) – Overground training.

Because the overall goal of the LTP is to achieve independent community walking within the range of normal walking speed using an optimal stepping pattern. The structured LTP, by training across two environments: the treadmill with a body weight support and treadmill (BWST) system (see Figure 2) [55] and overground (see Figure 3), targets the essential control and functional requirements of walking: (1) a reciprocal stepping pattern, (2) dynamic equilibrium during propulsion, and (3) adaptability to behavioral goals of the participant and environmental constraints.

The program consists of 20–30 minutes of step training using the BWST modality with manual assistance provided by trainers, followed by 15 minutes of overground assessment and ambulation training 3×/week for 36 sessions. A 20–30 min period of actual stepping is the goal for the intervention sessions on the treadmill with rest periods as needed. Each training session may last up to 1 hour and 30 minutes including time for warm-up, stretching, and cool down. The overall goal is to enable the participant, by the end of training, to walk independently a total of 20 minutes in four, 5 minute bouts at 0.89 – 1.2 m/s (2.0 – 2.8 mph) and 0% BWS with good stepping kinematics. Good stepping is defined as walking with (1) an upright trunk with pelvic rotation to achieve limb loading, (2) symmetrical stride length, (3) symmetrical swing and stance time, (4) hip and knee flexion moments during swing initiation and swing, and (5) hip and knee extensor moments during stance and push-off with proper stance and swing kinematics.

Parametric training targets include: (1) gradually decreasing BWS from 40% to 0%, (2) initiating treadmill speed in the range of normal walking speeds and increasing as tolerated, and (3) providing manual assistance initially when the participant is unable to independently step or control upright posture, and decreasing manual assistance to afford skill and control progression by the participant. One to three trainers assist the participants with verbal instructions and manual assistance to practice and achieve good stepping and posture. The progression of LTP training parameters during the course of the intervention are listed in Table 3. When bout duration of 5 minutes and total step time of 20 minutes are achieved, then the number of bouts may be decreased and the length of each bout increased. The target exercise capacity is 20 minutes of continuous, independent, good stepping on the treadmill at 0% BWS.

**Table 3:** Locomotor Training Progression

**Phase and Sessions**	**Training Priorities on the Treadmill**	**Treadmill (TM) Speed**	**Body-Weight Support**	**Intensity/Endurance**	**Manual Assistance**	**Overground/Community Ambulation**
Phase I (1–12)	1. Intensity 2. Speed 3. Independence at non-paretic leg	1.6 mph minimum (goal of 2.0 mph)	30 – 40%	Four 5-minutes bouts to attain 20 mins of stepping	As needed at trunk, paretic and nonparetic leg. Decrease assist at non-paretic leg.	1. Attempt skills observed/trained on the treadmill 2. Educate on use beyond clinic
Phase II (13–24)	1. Intensity at 2.0 mph and decrease BWS 2. Progress independence at trunk/pelvis with short bouts (decrease speed, increase BWS as needed)	2.0 – 2.8 mph	20% – 35%	Increase bout duration with decrease in number of bouts to attain 20 mins of stepping	Decrease assist to first trunk/pelvis, then paretic leg	1. Attempt skills observed/trained on the treadmill 2. Transfer skills/speed 3. Introduce assistive device and orthosis, as needed
Phase III (25–36)	1. Intensity to 30 mins 2. Progress independence with increased bout length, then increase speed, decrease BWS. 3. Adaptability (speed variations, stop/starts, incline)	2.0 – 2.8 mph and increasing speeds	0 – 20%	Increase total stepping time to 30 mins	Removed	1. Transfer skills/speed 2. Adaptability to environment: stairs, curbs, terrain and change speed, stops, turns.

Goals for each session address achieving training duration of 20 minutes, a minimum target training speed of 2.0 mph, and maximal body weight load while maintaining the kinematics and posture associated with walking. An early training priority is to achieve an upright symmetrical posture with spatial-temporal symmetry of the stepping pattern. Initially, the participant may walk with a shorter step length for the non-paretic limb and the step centered in the middle of the treadmill as a means to compensate for deficits in paretic limb and trunk control. A trainer thus initially works with the participant to verbally cue or manually assist with foot placement of the non-paretic limb.

The next phase of training continues repetitive practice of walking, but also provides opportunity for the participant to control his/her trunk and limb control. The priority is to gain trunk control first, then limb control. Achieving trunk/pelvic control is a necessity for overground walking. The speed may be decreased or BWS increased to allow independent practice of trunk control, limb control, or the entire stepping pattern. Errors will occur as participants tests their control abilities. As independence and control are achieved, the treadmill speed is increased and BWS decreased to continue to challenge the individual and to progress. As independence and control are achieved with minimal BWS and walking speed of 2.0 mph, then the last phase of training is introduced. Adaptability is challenged both in the treadmill and overground environments. Specifically, the participant adapts to conditions such as changes in the treadmill speed from slow to fast to slow again, sudden stops or starts, turning the head while walking, uneven terrain, or obstacles in the environment.

Step training using the BWST modality is followed by fifteen minutes of assessment and community ambulation training in the overground environment. The aim of community ambulation training is to transfer stepping capacity and skills from the BWST environment to overground walking. Participants stand and walk in the overground environment with guarding and/or manual assistance of trainers as transfer of stepping skills is assessed. Dimensions of stepping in the BWST environment that fail to transfer to overground walking are identified and appropriate instructions provided. The trainer and participant identify daily activities that support the overall goal of walking to be incorporated on a daily basis at home or in the community. Goals for the next treatment session using the BWST modality are set, based on the overground performance. Practice instructions for home may include use of an AFO or assistive device/support. As the stepping pattern and dynamic equilibrium improve and independence is achieved, the particular challenges of community ambulation are addressed by targeting performance of transitions (start and stopping), techniques to maximize endurance, walking over variable terrain, and negotiating obstacles. Evaluations and recommendations are made at the 12th, 24th, and 36th training sessions regarding specific use of assistive devices or braces.

For participants with poor proximal upper extremity muscle control, the hemiparetic arm may feel heavy and potentially painful during locomotor training. Additionally a heavy, flaccid upper-extremity can pull the trunk forward inhibiting good stepping. During training, the arm may be supported using a humeral cuff sling or with hand-hold support from a trainer. If shoulder range is available and voluntary movement is present and pain-free, armswing is encouraged.

#### Home Exercise Program

**Figure 4:**
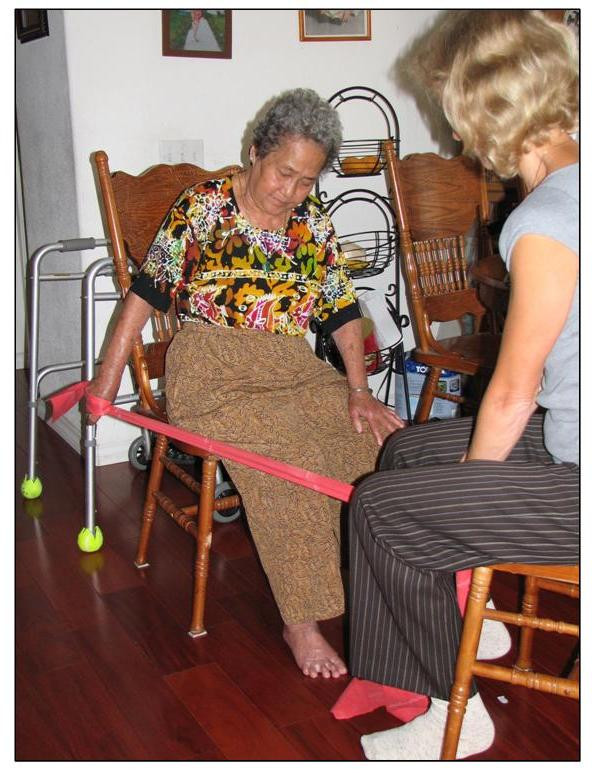
Home Exercise Program (HEP).

An exercise intervention designed to improve upper and lower extremity strength, sitting and standing balance, and coordination was designed for the home exercise group, incorporating techniques used in previous low-intensity and gait preparatory exercise programs (see Figure 4) [12-14]. The purposes of the home exercise program are to provide (1) an exercise-based intervention that is expected to have little or no effect on the primary outcome, gait speed, (2) an equal number of interactions and time spent with a physical therapist to minimize any potential for bias due to differential exposure and minimize the risk for differential loss to follow-up, and (3) a credible training program so that the participants would consider themselves involved in meaningful therapy activity. To match the LTP group, the home exercise group receives 36 therapy visits (3 times per week for 12 weeks) with length of HEP and LTP training sessions the same. Cardiovascular response monitoring during exercise is identical to that done in the LTP groups. In these ways, the HEP intervention will plausibly control for Hawthorne effect but exclusively through interventions that have been shown to have little or no impact on gait speed.

The exercise program is divided into three 4-week phases to provide the participants with a sense of progression. The first phase consists of upper extremity resistance exercise, lower extremity active exercises against no resistance, and sitting balance tasks. Each major joint of the upper and lower extremities is addressed. The second phase adds minimal resistance to the lower extremity, strengthening exercises, coordination tasks and static standing balance exercises. The third phase adds low repetitions of sit-to-stand practice and dynamic standing balance activities. At the end of each session, participants are encouraged to walk every day. Each participant is individually progressed according to their ability within each phase. Table 4 outlines examples of progression of exercises/activities. To ensure that all groups receive the same advice regarding the use of assistive devices for walking, the home exercise group participants are evaluated for assistive device needs after the 12th, 24th, and 36th session.

**Table 4:** Home Exercise Program

**Upper and Lower Extremity Exercises**	**Progression**
	Gravity eliminated, active assistive
	Gravity eliminated, active
	Against gravity, no Theraband
	Against gravity, yellow Theraband
	Against gravity, red Theraband
	Against gravity, green Theraband
	Against gravity, blue Theraband
	Against gravity, black Theraband
	Against gravity, silver Theraband

**Sitting Balance**	**Progression**

	Equal weight bearing on ischial tubersosities
	Weight shift, lifting opposite leg from chair
	Ipsilateral anterior diagonal reaching, NP* UE**
	Ipsilateral posterior diagonal reaching, NP UE
	Contralateral anterior diagonal reaching, P* UE
	Contralateral posterior diagonal reaching, P UE
	Contralateral anterior diagonal reaching, NP UE
	Contralateral posterior diagonal reaching, NP UE
	Ipsilateral anterior diagonal reaching, P UE
	Ipsilateral posterior diagonal reaching, P UE

**Static Standing Balance**	**Progression**

	Shoulder-width stance 30 sec, eyes open
	Shoulder-width stance 30 sec, eyes closed
	Feet together 30 sec, eyes open
	Staggered stance, P leg in front, eyes open
	Staggered stance, P leg in front, eyes closed
	Feet together 30 sec, eyes closed
	Staggered stance, P leg behind, eyes open
	Staggered stance, P leg behind, eyes closed
	Staggered stance, P leg in front on step, eyes open
	Staggered stance, P leg in front on step, eyes closed
	Staggered stance, NP leg in front on step, eyes open
	Staggered stance, NP leg in front on step, eyes closed

**Dynamic Standing Balance**	**Progression**

	Catching ball straight on
	Catching ball thrown towards NP side
	Catching ball thrown towards P side
	Turning towards P side
	Turning towards NP side

*P = paretic; NP = nonparetic

**UE = upper extremity

#### Vital Sign Monitoring for LTP and HEP Interventions

Blood pressure (BP) and heart rate (HR) are monitored prior to a session, during a session, and at the completion of each session. BP and HR must be within normal range for the participant prior to initiating each training session. HR must be less than 100 bpm to begin the training session. Participants' resting diastolic BP must be less than 100 and systolic BP less than 180 to begin the training session [56]. During the 20–30 minute training sessions, BP, HR, blood oxygen saturation, and the Borg scale Rate of Perceived Exertion are monitored every 5 minutes initially to assure that they remain within acceptable limits. The American College of Sports Medicine criteria for terminating an in-patient exercise session are followed according to guidelines shown to be effective for persons post-stroke with multiple comorbidities [56]. Persons that are on beta blockers are identified prior to initiating the training. Exercise tolerance for these individuals is specifically assessed using the Borg scale Rate of Perceived Exertion.

The criteria for termination of a training session include complaints of light-headedness or moderate or severe dyspnea, or the development of paleness and excessive sweating or confusion; complaints of feeling ill; onset of angina; pressure changes (systolic BP greater than 200 mm Hg, diastolic BP greater than 110 mm Hg), drop in systolic BP greater than 20 mmHG and inappropriate bradycardia (drop in heart rate greater than 10 beats per minute). In addition, should the participant's HR exceed 80% of the predicted maximum HR (220 – age) or the participant report a Borg exertion rate of greater than 12–13, then the training ceases. Should training be halted, the participant is asked to rest (sitting or standing) while BP and HR are monitored and training will resume only when vital signs have returned to within an acceptable range and excessive dyspnea or chest pain have resolved. If any of these conditions persist after rest, the patient's primary physician is called and the patient referred for evaluation. If the patient complains of angina at rest, loss of consciousness occurs, or cardiac arrest, emergency medical services through 911 are called immediately. All trainers are CPR certified and aware of signs of cardiac complications. All trainers and assistants are trained in procedures to afford quick and safe removal of a patient from the BWST system in the case of an emergency.

### Standardization for the Interventions

The LTP and HEP interventions are standardized to achieve consistent implementation of the intervention across clinical sites. Standardization assures that the training teams successfully implement a common intervention through application of six critical elements: (1) knowledge of the protocol; (2) goal setting, decision-making and progression; (3) participant safety and monitoring; (4) equipment use; (5) hands-on training skills; and (6) participant's role and participation. Documentation procedures are standardized across sites and require trainers to record in a computerized database all training parameters for the BWST and overground training.

A competency-based training program was used to train the trainers across all sites. The intervention teams met together for a 5-day training course, returned to their clinical sites for 2–3 months of pilot training, with follow-up sessions at each of the 5 sites to complete competency-based training and testing. Competency is required in each of the six knowledge and application domains and includes both written and practical components.

After individual therapists/trainers and the site achieve competency status, they are approved to admit participants to the RCT. Competency status is maintained throughout the trial by each trainer at every site and is systematically reviewed during the first participant's training after 1, 4, 8, and 11 weeks; then every 3 months by the Co-PIs (or an independent reviewer).

Turnover in trainers across sites is anticipated across a 3 year span of participant entry and training. The intervention site team leader is responsible for training new staff. The trainer, in accordance with her/his specific role and responsibilities in the trial, must achieve intervention competency before joining the site training team in treating enrolled participants. Any deviation from established competency standards requires immediate remediation and reevaluation. No trainer is allowed to conduct treatment without established and maintained competency. The Clinical Research Coordinator and co-PIs are responsible for maintaining standardization and competency throughout the trial.

Communication between site lead trainers, clinical research coordinators, and the co-PIs is maintained through weekly conference calls. A web-based, discussion board specific to each intervention provides timely responses to questions from the training teams with responses available to all training personnel. This list of questions and responses is recorded throughout the trial and used to refine or clarify the training manual. The Clinical Research Coordinators conduct weekly to bi-monthly visits to each site and relay any intervention-related concerns to the Co-PIs. As a follow-up to site visits or interim standardization reviews of training session videos and training session documentation, pertinent aspects of the HEP and LTP interventions are highlighted and discussed on conference calls to maintain consistency in decision-making and progression. Finally, the investigators have prepared an intervention training manual for therapists available on the LEAPS website. In addition, all participants received a Participant Manual for the LEAPS trial specific to either the HEP or LTP intervention to inform them of what to expect, goal-setting, progression across the sessions, and the role of the participant and trainers throughout the training.

### Statistical Analysis

#### Sample Size and Participant Accrual

LEAPS is powered to detect a difference of 20% in the proportion of participants achieving a successful locomotor recovery (defined as >0.4 m/s gait speed for participants with initial severe impairment, and >0.8 m/s for those with moderate impairment) between the locomotor training groups (LTP-early and LTP-late) and the HEP group, and a 0.1 m/s mean difference in gait speed change between the two locomotor training groups (LTP-early and LTP-late).

Power calculations indicate that a total of 340 participants are needed (120 in each of the two LTP training groups and 100 in the HEP group) in order to detect with 85% power a difference of 20% between each of the LTP treatment groups and the HEP group at the significance level = 0.05. In addition, with a sample size of 120 in each of the two LTP training groups, LEAPS will have 85% power to detect a difference of 0.1 m/s between each of the LTP groups using a two-sample t-test at the 0.05 significance level (two sided).

Because LEAPS is anticipating a 15% attrition rate at 1-year post-stroke, we plan to recruit and randomize 400 participants (140 in each of the two LTP training groups and 120 in the HEP group) in order to achieve our sample size goal. To achieve our recruitment goal of 400 participants from 5 sites in 2.5 years (32/site/year), LEAPS expects that for each year, each site will be able to train 24 participants with a BWST modality for 36 sessions.

#### Primary Statistical Analyses

##### Baseline

Demographic and baseline levels of clinical variables will be compared between the three randomized groups using analysis of variance (ANOVA) for comparison of means and chi-square tests for comparison of proportions. Variables found to be significantly different between the treatment groups will be considered in the endpoint analyses, in addition to the pre-specified covariates (e.g., severity, clinical site, age, stroke type, lesion location, and depression).

##### Specific Aims

###### Specific Aim 1

To determine if a structured locomotor training program (LTP) delivered early (2 months) or late (6 months) post-stroke is more successful in achieving clinically significant gains in locomotor recovery at 1-year post-stroke than the home exercise program (HEP) intervention.

###### Hypothesis 1

At 1-year post-stroke, there will be a clinically significant difference between each of the training groups (LTP-early, LTP-late) and the HEP group in the proportion of participants who successfully recover walking ability. Successful recovery of walking is defined as having achieved a 0.4 m/s gait speed or greater for persons with initial severe gait impairment (< 0.4 m/s) or as having achieved a 0.8 m/s gait speed or greater for persons with initial moderate gait impairment (≥ 0.4 m/s – < 0.8 m/s). A clinically significant difference will be defined as a greater than 20% difference in the proportion of participants who achieve successful recovery. We expect that both the LTP-early and LTP-late groups will have more success than the HEP group.

###### Analysis Plan

The purpose of this analysis is to test the hypothesis that there will be a clinically significant difference in successful recovery between the three randomized groups (LTP-early, LTP-late and HEP). First, we will provide summary statistics on the success proportions for each of the three groups by severity. Then, we will conduct a logistic regression with successful recovery of walking (yes/no) as the dependent variable, and two independent variables (*X*_1_, *X*_2_) indicating each of the training groups (LTP-early, LTP-late, and the HEP as the reference group). Other explanatory factors will include severity, clinical site, age, stroke type, lesion location, and depression. The odds ratios for achieving successful outcome for each treatment relative to the HEP group will be estimated. As indicated in the sample size section, the 20% difference in success proportions translates into an odds ratio of 2.33 for each treatment. Formally, if we let *p*_*ij *_denote the probability of successful recovery at one year post-stroke for the *j*^*th *^participant in the *i*^*th *^treatment group, and define *Z*_*ij *_as the baseline covariates for the corresponding participant, then the regression model can be written as follows: log *it*(*p*_*ij*_) = *α *+ *β*_1_*X*_1 _+ *β*_2_*X*_2 _+ *Z*_*ij*_*γ*. The Hochberg step-up procedure [xx] will be used to test two individual null hypotheses *β*_1 _= 0 and, *β*_2 _= 0, after controlling for severity and other covariates. Start with the larger p value *p*_(2)_, if *p*_(2) _≤ 0.05, then stop testing and reject both hypotheses thus we conclude that both LTP groups are significantly different from the HEP group; otherwise the LTP group corresponding to the larger p value is claimed to be not significantly different from the HEP and continue to check the smaller p value *p*_(1) _; if *p*_(1) _≤ 0.025, then the corresponding hypotheses is rejected and we conclude that the corresponding LTP group is significantly different from the HEP group; otherwise none of the two LTP groups is significantly different from the HEP group. In addition, we will perform post-hoc tests to determine whether the differences occurred in the severely impaired participants, the moderately impaired participants, or both. The particular value of using this logistic regression approach to test this hypothesis is that it allows us to put all independent variables of potential importance into the model without making any a priori assumptions about the presence or absence of time of treatment effects, or effects related to clinical site, age, stroke type, lesion location, or presence of depression. LTP can be justified only if a clinically significant difference between LTP and HEP participants can be demonstrated. Only when such a difference has been established does it make sense to ask when the treatment should take place and whether severity of impairment influences optimal timing of treatment (the foci of specific Aim 2).

###### Specific Aim 2A

To determine if the timing of LTP delivery (early vs. late) affects the improvement in gait speed at 1-year post-stroke.

###### Hypothesis 2A

The improvements of gait speed from baseline to 1-year post-stroke for participants trained at 2 months will be significantly greater than for participants trained at 6 months

###### Specific Aim 2B

To test whether initial locomotor impairment severity interacts with the timing of LTP delivery to affect the improvement in gait speed at 1-year post-stroke.

###### Hypothesis 2B

The timing of LTP delivery will interact with baseline locomotor impairment severity such that (1) individuals with more severe initial walking impairment will demonstrate greater improvements in gait speed from LTP-late than LTP-early and (2) individuals with less severe initial walking impairment will demonstrate greater improvements in gait speed with LTP-early than LTP-late.

##### Analysis Plan

The rationale behind this analysis is that, once a clinically significant difference between the LTP and HEP groups has been established (Specific Aim 1), then any statistically significant difference in timing effect or in timing by severity interaction effect will become clinically relevant. If at least one of the two individual null hypotheses is rejected in the analysis for Specific Aim 1, we will conduct formal statistical inference to compare the two treatment groups (trained during the sub-acute or chronic periods post-stroke) based on improvement in gait speed from 2-month baseline assessment to 1-year post-stroke. This second primary analysis will fit a linear model for the gains of gait speed. The explanatory factors will include study group, severity, clinical site, age, stroke type, lesion location, and depression. Let *Y*_*ij *_denote the improvement in gait speed at 1-year follow up for *j*^*th *^the participant in the *i*^*th *^treatment by severity group, where *i *= *ES*, *LS*; *EM*, *LM*. (ES, early severe; LS, late severe; EM early moderate; LM, late moderate). We define *Z*_*ij *_as the baseline covariates for the corresponding participant. The linear model can be written as follows: *Y*_*ij *_= *α*_*i *_+ *Z*_*ij*_*β *+ *ε*_*ij*_. In this model, *α*_*i *_is the mean of gait speed improvement within *i*^*th *^treatment by severity group and *β *is the covariates effect on response. The error term *ε*_*ij *_is assumed to have normal distribution with zero mean and variance *σ*^2^. Similar to the Specific Aim 1, we will employ the Hochberg step-up procedure [xx] to test two individual null hypotheses *α*_*ES *_= *α*_*LS *_and *α*_*EM *_= *α*_*LM*_, with the larger p value compared to 0.05 and the smaller p value compared to 0.025. If the null hypothesis *α*_*ES *_= *α*_*LS *_is rejected, we would conclude that the timing of intervention delivery affects mean 1-year outcome for the severe group. Similarly, we may conclude *α*_*EM *_≠ *α*_*LM *_which indicates a timing effect for the moderate group. In addition, we would perform post-hoc tests to determine whether the timing effects are the same for the two severity levels.

#### Secondary Statistical Analyses

##### Specific Aim 3A

For each LT group, to determine if the number of locomotor training sessions affects the outcome of gait speed immediately following the 12th, 24th, and 36th sessions.

##### Hypothesis 3A

There will be a significant increase in gait speed for participants when assessed following increasing numbers of training sessions, such that gait speed after the 24th session will be greater than after the 12^th ^session and gait speed after the 36th session will be greater than after the 24th session.

##### Specific Aim 3B

To test whether initial locomotor severity interacts with the number of treatment sessions to affect the gait speed immediately following the 12th, 24th, and 36th sessions.

##### Hypothesis 3B

The number of locomotor training sessions will interact with baseline locomotor impairment severity such that (1) individuals with more severe initial walking impairment will demonstrate greater improvements in gait speed from an increasing number of training sessions and (2) individuals with less severe initial walking impairment will not benefit from more extended training.

##### Analysis Plan

For each LTP group, a longitudinal analysis will be conducted using gait speed before the training and following the 12th, 24th, and 36th sessions, taking into consideration the dependence of repeated measurements at different time points for each participant. The model will include baseline impairment severity, categorical assessment time and their interactions. It will be performed using the MIXED procedure of SAS software. The regression coefficient associated with assessment time represents the average rate of change in gait speed. Treatment group differences in the estimated gait speed change rate will be tested.

#### Tertiary Statistical Analysis

Additional analyses will assess the differences in improvements between the three groups in the Fugal-Meyer Motor Scores, the Berg Balance Score, distance walked in 6 minutes, community ambulation (as measured by the step activity monitor), and self-reported quality of life (as measured by the Stroke Impact Scale -Participation domain). We will also assess differences in the incidence of depression between the three groups. A multivariate ANOVA (or ANCOVA) will be conducted to compare the 2-month to 12-month change scores between the three groups: LTP-early, LTP-late and HEP. If there are significant differences among the three groups, then linear contrast tests will be employed to detect pairwise differences. We will conduct a logistic regression with incidence of depression (yes/no) as the dependent variable. In addition, a Poisson regression will be conducted to assess whether the intervention reduces the number of falls. Lastly, a regression analysis will be conducted with gait speed change from 2-month to 12-month post-stroke as dependent variable for the HEP group. The explanatory factors will include severity, clinical site, age, stroke type, lesion location and depression.

#### Missing data

For participants who drop out and cannot complete the one-year evaluation, we will impute outcome based on the last available assessment value, with the proviso that, for those who dropped out due to a related adverse event, the dichotomous outcome will be imputed as failure in the logistic regression analysis and the improvement in gait speed as the minimum of zero and the change from the baseline and last assessment in the linear regression analysis. More precisely, the change in gait speed will be defined as Δ = min(0, *g*_*lastobs *_- *g*_*lastobs*_) for those who dropped out due to a related adverse event, and as Δ = *g*_*lastobs *_- *g*_*lastobs *_for all other "non-completers"; the successful recovery of walking (yes/no) is defined correspondingly.

In addition, we will perform sensitivity analyses by comparing results from the intent-to-treat analyses described above with the subgroup of participants with one-year follow-up data, as well as those obtained using different imputation procedures, including: (1) missing one-year outcome predicted by participant gait speed trajectory; (2) missing one-year outcome predicted by participant gait speed trajectory, plus baseline demographic and clinical factors, and (3) missing one-year outcome predicted by a model that takes into account participant dropout bias. For this last model, we will evaluate participant dropout bias through the following four steps: (1) determine demographic and clinical variables that characterize differences between "completers" and "non-completers"; (2) develop a model predicting outcomes for the "completers" using the significant independent variables from the previous step; (3) use the resulting model to predict outcomes for the non-completers; and (4) redo the primary analyses for the full dataset.

### Adverse Event Monitoring and Reporting

Adverse events are carefully monitored at every level of the LEAPS trial. The teams at the clinical intervention sites monitor and report all minor and serious adverse events that occur with any participant from the point of enrollment through the 12-month final follow-up assessment.

The LEAPS trial is supported by an NIH appointed Medical Safety Monitor, independent of the study executive committee, who reviews all serious adverse events. An NIH appointed Data Safety Monitoring Board provides oversight and meets biannually. All adverse events are reported to the DSMB every 6 months.

The LEAPS clinical intervention sites report adverse events to their local Institutional Review Boards (IRBs). All serious, unexpected and related events at any site are reported to every IRB within one week. Some of the site IRBs require reporting of all serious adverse events, regardless of whether or not they were expected or related, within the one week timeframe. Other site IRBs allow for the reporting of expected and/or unrelated serious adverse on a yearly basis. All minor adverse events are reported to the site IRBs once a year at the time of renewal of IRB approval.

#### Definitions for adverse event monitoring

##### Serious Adverse Events

Using general IRB guidelines the LEAPS trial has specifically defined what constitutes a serious adverse event: The following events are considered serious: death, life-threatening adverse event (stroke, MI, fracture), inpatient hospitalization, a persistent or significant disability or incapacity that lasts more than 48 hours and limits activities of daily living.

Serious adverse events are reviewed to determine whether or not the event is expected. An expected adverse event is one that is part of the normal disease progression or one that is listed in current investigator brochure, protocol, or informed consent form. The initial determination of whether or not an event is expected is made by the site medical director. The medical safety monitor also judges whether or not the event is expected.

The LEAPS trial also considers whether or not an event is related to the trial. A "related" adverse event reflects a realistic chance of a causal relationship between the study intervention and the adverse event, as suggested when an event occurs within a short time after the intervention (i.e., 24 hours), follows a pattern consistent with the study intervention, and improves when the study intervention has stopped and/or reappears when the intervention is resumed. As with expectedness, whether or not an event is related to the screening, exercise tolerance test, the study assessments or the study intervention is initially judged by the site medical director. The medical safety monitor also judges whether or not the event is related to any of the study procedures. Information on whether or not an event is deemed related to the LEAPS trial is forwarded to the Institutional Review Boards according to their individual regulations.

In some cases a relationship with the trial can not be ruled out. Such a relationship exists when the reaction follows a pattern consistent with the study intervention and improves when the intervention is stopped, but could have been caused by the condition being treated or by other interventions. This is also judged by the site medical director and the medical safety monitor.

##### Minor Adverse Events

The following events are considered minor adverse events: (1) Fall with no fracture, (2) Dyspnea, (3) Open sore or blister, cuts (break of skin), (4) Muscle soreness or pain that persists for more than 48 hours, (5) Dizziness/fainting, (6) Diaphoresis, (7) hypertension during exercise that requires stopping the intervention for the day, (8) Low blood pressure that requires stopping the intervention for the day, (9) Deep venous thrombosis. Minor adverse events are reported to the site IRBs once a year at the time of renewal.

##### Reporting Procedures

The LEAPS trial utilizes a web-based data entry system and all Adverse Events are input into this system as soon as the site team becomes aware of the event. The LEAPS data entry system then employs an automated notification system that sends an email regarding all serious and minor adverse events to the site team leaders, the site research assistants, the clinical research coordinators, the co-principal investigators, the principal investigator, the project manager and the medical safety monitor.

When an adverse event occurs, the site team leader immediately fills out an initial adverse event report. If the event is minor, a second minor adverse event form is completed. In the case of a serious adverse event, the team notifies the site medical director immediately and then confirms the event within 3 business days. Once the event is confirmed as a serious adverse event, the team, including the site medical director, completes a serious adverse event confirmation form. Serious adverse events are confirmed by review of medical records or by contacting the participant's primary care physician.

The Medical Safety Monitor fills out a report adjudicating the event and determining whether the event is, in his opinion, related to the trial. The Medical Safety Monitor also provides a recommendation as to whether the participant should remain in the trial. All sites have online access to the completed adverse event reports and are responsible for reporting the events to the individual Institutional Review Boards that oversee the clinical intervention sites.

The site's Medical Director, the study PI and the Medical Safety Monitor will review all serious adverse events immediately and determine if the participant is to be discontinued from the trial. If the adverse event is related and precludes continuation, the participant will be officially dropped from the study. If the adverse event is unrelated or a temporary condition and the patient is medically cleared to continue in the trial, she/he will not be dropped.

The Data Management and Analysis Center provides monthly reports of all serious adverse events. These reports are sent to each site and to the administrative coordinating center for review. This allows for the review of all adverse events and to ensure that all events are followed to conclusion and that participants are appropriately maintained or discontinued from the study.

### Data Management and Quality

#### Data Management and Quality Control Procedures

The Data Management and Analysis Center (DMAC) has developed and implemented a secure LEAPS database and web-based data entry system. The LEAPS database system facilitates data entry with built in quality control checks (e.g., range checks, checking for missing data for required data points). In addition, the LEAPS web-based system provides (1) a public website including study information, background and responsibilities of key personnel, news items, employment opportunities, publications, and (2) a secure website information housing the Manual of Procedures, data collection forms, reports to the Steering Committee, minutes of conference calls with study investigators, reports and updates on recruitment status, reporting of SAEs, and reports to the Data Safety and Monitoring Board (DSMB).

Additional quality control procedures include: (1) randomly selected comparison of data points between the paper records and the electronic records in the SQL database at each study site, and (2) frequency distributions checks of key outcome variables (overall, not stratified by treatment assignment) along with a list of participant IDs associated with possible outliers or questionable data points. Quality control summary reports are also housed on the secure LEAPS website.

### Study Organization and Management

The LEAPS Trial is managed by an executive steering committee. This committee is responsible for the overall trial management including oversight of participant recruitment, execution of the trial, ethical conduct of the trial, study publications and ancillary studies. The steering committee includes the Principal Investigator Dr. Pamela Duncan, the Co-principal Investigators, Dr. Andrea Behrman, and Dr. Kathy Sullivan. Dr. Stephen Nadeau and Dr. Bruce Dobkin are the study Neurologists and Neurorehabilitation consultants. Dr. Stanley Azen is the Director of the Data Management and Analysis Center and Dr. Sam Wu is the study lead statistician. Ms. Sarah Hayden is the Project Manager. Dr. Duncan, the study principal investigator chairs the committee. The executive steering committee has conference calls every week and meets once a year in person. There is a subcommittee of clinical site medical directors. This committee has a monthly phone call with the executive steering committee. Each clinical intervention site receives oversight from a Clinical Research Coordinator. The Clinical Research Coordinators for this trial are Julie Tilson, DPT and Dorian Rose, Ph.D., P.T. The Clinical Research Coordinators have a weekly conference call with the site team leaders and visit the clinical facilities at least on a biweekly basis. The clinical research coordinators and the study principal and co-principal investigators have weekly conference calls to ensure that all assessment, intervention, and patient recruitment issues are dealt with in an efficient and consistent manner across sites.

There is an NINDS appointed Data Safety Monitoring Board (DSMB) to oversee the trial. The DSMB is responsible for assuring NINDS that the study is safe and conducted according to high scientific and ethical standards. The DSMB assesses participant recruitment, retention and follow-up, and data quality. The DSMB also reviews all adverse events and monitors safety issues. It reviews all proposed protocol changes and all ancillary study proposals. Then DSMB meets with Dr. Duncan, Dr. Azen, Dr. Wu, and an NINDS representative twice a year. A summary of the DSMB meeting and subsequent recommendations are forwarded to NINDS, the principal investigator, and respective IRBs.

This trial also has an NINDS appointed Medical Safety Monitor, who is independent of the trial investigators and reviews all serious adverse events as they occur.

The DMAC is co-directed by Dr. Stanley Azen, Dr. Steven Cen and Dr. Samuel Wu. The co-directors oversee the operation of the DMAC, including data management and analysis of all data sources. Dr. Wu is responsible for conducting the primary statistical analyses for the Specific Aims, reviewed by Drs. Azen and Cen. All three Co-directors will be responsible for running secondary and tertiary statistical analyses.

The Co-directors are assisted by the Bioinformaticist and Web Manager (James Baurley MS and James Gardner), who developed and oversee the LEAPS database system and website. In addition, Dr. Cen interacts with the Database Manager (Chris Hahn, MS) in producing recruitment, follow-up and compliance reports, quality control reports, and biannual Data and Safety Monitoring Board (DSMB) reports. Dr. Cen also oversees the training process of LEAPS study personnel in using the database system, the randomization process, and the reporting of SAEs.

The DMAC members meet biweekly and two executive committee calls each month are lead by Stan Azen. During these calls Dr. Azen reports on all data acquisition, data management, and analysis issues to the executive committee.

## Discussion

This multi-site RCT is designed to determine the impact of LTP compared to a home exercise program on clinically and functionally meaningful changes in function for patients with acute and sub-acute stroke. Stroke results in disabling limitations for patients. These patients turn to rehabilitation specialists for evidence-based care that will maximize their recovery. The results of this RCT will provide a significant addition to our body of knowledge regarding LTP post-stroke and more broadly to our understanding of how to conduct large scale, multi-site clinical trials of rehabilitation interventions.

This trial will make several unique contributions to the practice of locomotor therapy after stroke. First and foremost, it will test the value of a clinically practicable locomotor training program in a sufficient number of participants of sufficient variety to provide a reasonable basis for confident inference to clinical practice. The primary endpoint of the trial is clinically relevant and measured one year post-stroke to assesses the sustainability of any immediate post treatment benefit.

The value of LTP will be tested against a home exercise program (HEP) intervention that not only incorporates usual and customary care but also provides a sufficiently intensive intervention to provide a plausible control for Hawthorne effect. The trial will help to determine the optimal timing of intervention. Optimal timing of neurorehabilitation remains a subject of scientific dispute and even precisely controlled animal studies have not settled the issue  [57]. The trial will provide a start in the process of understanding the optimal dose of treatment for any given participant. Finally, the trial will examine the impact of treatment on a sufficient number of ancillary functional measures to make its outcomes readily comparable with those of other trials.

**Current study status: **Enrolling Patients

## Abbreviations

RCT – Randomized Controlled Trial; LTP – Locomotor Training Program; HEP – Home Exercise Program; BWST – Body Weight Support and Treadmill; BWS – Body Weight Support; DMAC – Data Management and Analysis Center; DSMB – Data Safety Monitoring Board; SAE – Serious Adverse Event

## Competing interests

The author(s) declare that they have no competing interests.

## Authors' contributions

PWD led the conceptualization, design, and implementation of this research protocol. She was the primary author for this manuscript.

KJS was a leader in the conceptualization, design, and implementation of this research protocol. She is a contributing author for this manuscript.

ALB was a leader in the conceptualization, design, and implementation of this research protocol. She is a contributing author for this manuscript.

SPA led the development of the data management protocol and statistical analysis plan. He is a contributing author for this manuscript.

SSW has been a leader in the development of the analysis plan and was a leader in the conceptualization and design of this research protocol. He is a contributing author for this manuscript.

SEN and BHD assisted in the design of this protocol.

DKR and JKT assisted in the design of the protocol for interventions and assessments, as well as all data collection procedures. They contributed to writing of this manuscript.

All authors reviewed and approved this manuscript prior to submission.

## Pre-publication history

The pre-publication history for this paper can be accessed here:


